# Salmon Gravlax Biopreservation With Lactic Acid Bacteria: A Polyphasic Approach to Assessing the Impact on Organoleptic Properties, Microbial Ecosystem and Volatilome Composition

**DOI:** 10.3389/fmicb.2019.03103

**Published:** 2020-01-21

**Authors:** Norman Wiernasz, Françoise Leroi, Frédérique Chevalier, Josiane Cornet, Mireille Cardinal, Jens Rohloff, Delphine Passerini, Sigurlaug Skırnisdóttir, Marie-France Pilet

**Affiliations:** ^1^Laboratoire Ecosystèmes Microbiens et Molécules Marines pour les Biotechnologies (EM3B), Ifremer, Nantes, France; ^2^UMR 1014 SECALIM, INRA, Oniris, Nantes, France; ^3^NTNU, Department of Biology, Norwegian University of Science and Technology, Trondheim, Norway; ^4^Matıs, Research and Innovation, Exploitation and Utilization of Genetic Resources, Reykjavik, Iceland

**Keywords:** metabarcoding, 16S rRNA gene, seafood, antilisterial activity, volatile organic compounds, sensory analyses

## Abstract

Seafood and fishery products are very perishable commodities with short shelf-lives owing to rapid deterioration of their organoleptic and microbiological quality. Microbial growth and activity are responsible for up to 25% of food losses in the fishery industry. In this context and to meet consumer demand for minimally processed food, developing mild preservation technologies such as biopreservation represents a major challenge. In this work, we studied the use of six lactic acid bacteria (LAB), previously selected for their properties as bioprotective agents, for salmon dill gravlax biopreservation. Naturally contaminated salmon dill gravlax slices, with a commercial shelf-life of 21 days, were purchased from a French industrial company and inoculated by spraying with the protective cultures (PCs) to reach an initial concentration of 10^6^ log CFU/g. PC impact on gravlax microbial ecosystem (cultural and acultural methods), sensory properties (sensory profiling test), biochemical parameters (pH, TMA, TVBN, biogenic amines) and volatilome was followed for 25 days of storage at 8°C in vacuum packaging. PC antimicrobial activity was also assessed *in situ* against *Listeria monocytogenes*. This polyphasic approach underlined two scenarios depending on the protective strain. *Carnobacterium maltaromaticum* SF1944, *Lactococcus piscium* EU2229 and *Leuconostoc gelidum* EU2249, were very competitive in the product, dominated the microbial ecosystem, and displayed antimicrobial activity against the spoilage microbiota and *L. monocytogenes.* The strains also expressed their own sensory and volatilome signatures. However, of these three strains, *C. maltaromaticum* SF1944 did not induce strong spoilage and was the most efficient for *L. monocytogenes* growth control. By contrast, *Vagococcus fluvialis* CD264, *Carnobacterium inhibens* MIP2551 and *Aerococcus viridans* SF1044 were not competitive, did not express strong antimicrobial activity and produced only few organic volatile compounds (VOCs). However, *V. fluvialis* CD264 was the only strain to extend the sensory quality, even beyond 25 days. This study shows that *C. maltaromaticum* SF1944 and *V. fluvialis* CD264 both have a promising potential as bioprotective cultures to ensure salmon gravlax microbial safety and sensorial quality, respectively.

## Introduction

Originally from Scandinavia, and widely consumed in Nordic countries, fish gravlax products are gaining popularity elsewhere in Europe (FranceAgriMer, 2018). These products, mainly based on fish such as salmon (*Salmo salar*), whitefish (*Coregonus lavaretus*), rainbow trout (*Oncorhynchus mykiss*), herring (*Clupea harengus*) and mackerel (*Scomber scombrus*) (Leisner et al., 1994; Lyhs et al., 2001), are now commonly found on refrigerated supermarket shelves as ready-to-eat products. Although there are as many recipes as chefs, fish gravlax is usually prepared by curing fish filets in a mixture of dry salt-sugar base, to which are often added spices such as dill and black pepper, with no pre-heating treatment or smoking process. They are mainly characterized by a salt content of 3–6% and by a pH of 5–6 (Morzel et al., 1999; Lyhs et al., 2001). Commercial products are stored chilled, often sliced and vacuum-packed.

Considering the technologically simple processes used for its preservation (salting, vacuum-packaging and cool storage), fish gravlax is considered a lightly preserved fish product (LPFP), with a shelf-life often not exceeding 18–27 days (Leisner et al., 1994; Lyhs et al., 2001). Fish gravlax products offer an ideal growth environment for psychrotrophic pathogenic bacteria or specific spoilage microorganisms involved in sensory degradation (Gram and Huss, 1996). The microbial ecosystem of LPFP stored in vacuum–packaging is usually dominated by psychrotrophic Gram-negative spoilage bacteria such as *Photobacterium* spp. (*P. phosphoreum*, *P. illiopiscarium*), *Vibrio* spp., *Shewanella* spp. (*S. baltica*, *S. putrefaciens*), but also *Enterobacteriaceae* such as *Serratia proteamaculans* and *Hafnia alvei*, which can easily reach 10^7^–10^8^CFU/g during storage (Paludan-Müller et al., 1998; Lyhs et al., 2001; González-Rodrìguez et al., 2002; Cardinal et al., 2004; Leroi, 2014). *Brochothrix thermosphacta*, and many lactic acid bacteria (LAB) such as *Carnobacterium* spp. (*C. maltaromaticum*, *C. divergens*), *Lactobacillus* spp. (*L. farciminis*, *L. alimentarius*, *L. plantarum*, *L. delbrueckii*, *L. sakei*), *Leuconostoc* spp. (*L. mesenteroides*, *L. gelidum*), *Vagococcus* spp. (*V. fluvialis*, *V. penaei*) and *Weissella* spp. can also be found as dominant microbial group and may contribute to spoilage (Leisner et al., 1994; Morzel et al., 1999; Lyhs et al., 2001, 2002; Leroi, 2010, 2014; Pilet and Leroi, 2011). Moreover, like many LPFPs such as smoked fish, marinated fish, seafood salads and lightly cooked products, fish gravlax products also represent a high potential risk for listeriosis transmission (Cruz et al., 2008; Pilet and Leroi, 2011; Jami et al., 2014), and have the highest level of non-compliance to *Listeria monocytogenes* criteria in Europe (EFSA and ECDC, 2018).

To face today’s consumer demand for minimally processed high quality food, without chemical additives, new trends such as biopreservation and phage biocontrol have appeared as promising green solutions (Ghanbari et al., 2013; Le Fur et al., 2013). As defined by Stiles (1996) biopreservation concerns the use of microorganisms or their metabolites to extend shelf-life and enhance the safety of food commodities. Possessing antimicrobial properties and as natural dominant microbiota of many food products, LABs are now widely studied for the biopreservation of fruits, fermented and raw vegetables, dairy and bakery products, meat and seafood (Pilet and Leroi, 2011; Ghanbari et al., 2013; Zagorec and Christieans, 2013; Axel et al., 2017; Leyva Salas et al., 2017; Singh, 2018).

Most of the scientific work on seafood biopreservation focuses mainly on microbial safety control, especially for *L. monocytogenes* growth inhibition (Ghanbari et al., 2013). For that purpose, bacteriocin-producing LABs, such as *Carnobacterium* species (*C. maltaromaticum* and *C. divergens*) and *L. sakei* have been particularly well-studied (Pilet and Leroi, 2011).

Compared to microbial safety control, biopreservation for seafood shelf-life extension is far less documented. Spoilage is a complex process that may involve several microorganisms and often needs to be assessed by a polyphasic approach combining microbiological and biochemical analyses (pH, TVBN, TMA, PV measurement) and sensory evaluation. For instance, through the application of *L. piscium* strain CNCM I-4031, Matamoros et al. (2009) and Fall et al. (2010a), greatly improved the sensory shelf-life of cold-smoked salmon and cooked and peeled shrimp, respectively.

In previous work, six protective cultures (PCs): *Carnobacterium maltaromaticum* SF1944, *Lactococcus piscium* EU2229, *Leuconostoc gelidum* EU2249, *Vagococcus fluvialis* CD264, *Carnobacterium inhibens* MIP2551 and *Aerococcus viridans* SF1044, were screened and selected as promising PCs for seafood (Wiernasz et al., 2017). The present work set out to study the use of these six PCs for salmon dill gravlax safety and quality improvement. Their biopreservation potential was assessed with an approach combining cultural microbial analyses, sensory evaluation and biochemical analyses (pH, TMA, TVBN, biogenic amines). Volatilome characterization was also done to potentially reveal the metabolic activity performed by the applied PCs. Their growth in the products and their impact on the gravlax endogenous microbial ecosystem was also monitored by metabarcoding. To the best of our knowledge, this is the first study using such a polyphasic approach to monitor the effect of bioprotective LAB addition in food. This is also the first report on the biopreservation of fish gravlax, which differs from other seafood in its sucrose content, but also on the use of LAB species such as *V. fluvialis*, *C. inhibens*, and *A. viridans*.

## Materials and Methods

### Gravlax Production and Storage

Gutted salmons (*Salmo salar*) from the same batch were purchased in Norway and processed at the same industrial site in France. Salmons were fileted and cured with a mix of dry salt, sugar, pepper and dill for 14 h at 6°C. Filets were then rinsed, sliced and vacuum-packed as 120 g portions of 8–10 slices. After conditioning, salmon gravlax portions were transported to the laboratory under refrigerated conditions and then stored at 0°C until the start of the experiment (within 24 h).

### Bacterial Strains and Media

Information on the six PCs and *L. monocytogenes* strains is provided in Table 1. PCs and *L. monocytogenes* strains were stored at −80°C in their growth medium supplemented with 10% of sterile glycerol (Sigma-Aldrich, Steinheim, Germany). From the frozen stock, they were respectively subcultured in Elliker broth (Biokar Diagnostic, Beauvais, France) and Brain Heart Infusion broth (Biokar Diagnostic, Beauvais, France) for 48 h at 15°C before experiments.

**TABLE 1:** Protective cultures and *L. monocytogenes* strains.

**Species**	**Strains code**	**Origin**
**Protective cultures**		
*Carnobacterium maltaromaticum*	SF1944	Cold smoked salmon
*Lactococcus piscium*	EU2229	Fresh salmon
*Leuconostoc gelidum*	EU2249	Fresh salmon
*Vagococcus fluvialis*	CD264	Cooked and peeled shrimp
*Carnobacterium inhibens*	MIP2551	Fresh salmon
*Aerococcus viridans*	SF1044	Cold smoked salmon
**Pathogenic bacteria**		
*Listeria monocytogenes*	RF191	Shrimp
	RF107, RF114,	Cold smoked salmon
	RF131, RF151,	

*Strains from the collection EM^3^B Ifremer/SECALIM INRA-Oniris.*

### Gravlax Inoculation

#### PC Effect on Gravlax Quality

In the first trial (Batch 1), PC strains were precultured in Elliker broth medium for 48 h at 15°C before the experiment and were then diluted in tryptone salt broth (Biokar Diagnostic) to reach 10^8^ CFU/ml. To eliminate the growth medium, diluted suspension was pelleted by centrifuging for 10 min at 8,000 × *g* and pelleted bacterial cells were homogenized in the same volume of a new tryptone salt solution. Gravlax slices were inoculated at 2% (v/w) (1% on each side) with each PC strain separately by spraying, using an airbrush (Paasche airbrush H202S model, Paasche Airbrush Company, Chicago, IL, United States) to obtain an initial concentration in the product of around 10^6^ CFU/g. Slices were then vacuum-packed and stored at 8°C for 25 days. Non-inoculated gravlax packed portions were used as negative controls. Except for sensory analysis, all measurements were made in triplicate (biological replicates). Day T0 was set as the start of the experiment when gravlax was inoculated.

#### PC Effect on *L. monocytogenes* Growth

In the second trial (Batch 2), PC antilisterial activity was determined *in situ* against a cocktail of five *L. monocytogenes* strains (Table 1) on another salmon gravlax batch from the same company and produced in the same conditions. *L. monocytogenes* strains were cultivated individually in Brain Heart Infusion broth for 48 h at 15°C. Cultures were then pooled and diluted to reach a concentration of 10^6^ CFU/ml in tryptone salt broth. PCs were cultivated and diluted as described in Section “PC Effect on Gravlax Quality.” Dill gravlax slices were first inoculated at 1% (v/w) on only one side with *L. monocytogenes* strains suspension to reach a concentration of 10^4^ CFU/g. To allow bacterial adhesion, inoculated slices were stored 1 h at 4°C. Gravlax slices were then co-inoculated with PC diluted suspension at 1% (v/w) on the same side to reach an initial count of 10^6^ CFU/g. Gravlax slices inoculated with *L. monocytogenes* alone were used as controls. Slices were stored in vacuum packaging at 8°C for 21 days. Experiments were performed in triplicate.

### Microbiological Analysis

#### Bacterial Enumeration

Total viable count (TVC), LAB, *Enterobacteriaceae* and *Brochothrix thermosphacta* were enumerated for Batch 1 at T0 and after 7, 14, 18, 21, and 25 days by the culture method. At each sampling time, 20 g of product was aseptically withdrawn and stomached (Stomacher 400 circulator, Seward Medical, London, United Kingdom) for 2 min with 80 ml of refrigerated sterile tryptone salt solution (Biokar Diagnostic) with 1% Tween 80 (Grosseron, Saint-Herblain, France). Prior to dilution, the stomached solution was left at room temperature for 30 min for bacterial revivification. Successive dilutions were performed in tryptone salt solution with Tween, 1 ml of the appropriate dilution was pour-plated for *Enterobacteriaceae* enumeration, and 100 μl was spread-plated for the other microorganisms. For the different bacterial groups enumerated, culture media and growth conditions are listed in Table 2. To set anaerobic condition, Anaerocult A and Microbiology Anaerotest (Merck, Darmstadt, Germany) were added to hermetic jars. Detections thresholds were 0.7 and 1.7 log CFU/g, respectively, for *Enterobacteriaceae* and other counts.

**TABLE 2:** Culture media and growth conditions used for the enumeration of bacterial groups in gravlax samples from the Batch 1.

**Target bacterial group**	**Selective medium**	**Growth conditions**
Total viable count	Long and Hammer (LH)	96 h at 15°C, aerobic
Lactic acid bacteria	Nitrite Actidione Polymyxin agar (NAP)	96 h at 20°C, anaerobic
*Brochothrix thermosphacta*	Streptomycin-Thallous Acetate Actidione agar (STAA)	48 h at 20°C, aerobic
*Enterobacteriaceae*	Violet Red Bile Glucose agar (VRBG)	48 h at 20°C, double layer, aerobic
*Listeria monocytogenes*	Palcam agar	48 h at 37°C, aerobic

For Batch 2, *L. monocytogenes* and LAB were enumerated at T0 and after 14 and 21 days, by spread-plating on Palcam (Biokar Diagnostic) and NAP medium respectively. LABs and *L. monocytogenes* were incubated as described in Table 2.

#### Total Bacterial DNA Extraction

Bacterial DNA was extracted from stomached solution obtained for Batch 1 following a modified and optimized protocol using a MasterPure^TM^ Gram-positive DNA purification kit (Epicentre, Illumina, Madison, WI, United States). Four ml of stomached solution was spun down at 12,000 × *g* for 10 min at 4°C. After centrifugation, the supernatant was removed, and the cell pellet was re-suspended in 500 μl of TE buffer and treated with 1 μl of Ready-Lyse-Lysozyme for 1 h at 37°C with homogenization every 20 min. After lysozyme treatment, the mixture was transferred into tubes containing 0.2 g of sterile 1.0 mm zirconia/silica beads (Biospec Products, Bartlesville, OK, United States). Tubes were shaken twice for 2 min at 30 Hz with a bead beater (Retsch, Illkirch, France) with ice cooling between each cycle. Supernatant was then treated with 150 μl of Gram-positive lysis solution containing 1 μl of proteinase K (50 μg/μl) for 15 min at 65°C. After incubation, samples were cooled at 37°C for 5 min and then in ice for another 5 min. 175 μl of MPC protein precipitation reagent was added to the samples followed by vigorous shaking for 10 s, and centrifugation at 4°C for 10 min at 10,000 × *g*. Supernatant was treated with 1 μl of RNase A (5 μg/μl) for 1 h at 37°C. Five hundred μl of cold isopropanol (Carlo ERBA Reagents, Val-de-Reuil, France) was then added to the solution, which was homogenized by inverting tubes 40 times before overnight incubation at −20°C. The DNA pellet was recovered by centrifugation at 4°C for 10 min at 10,000 × *g* and rinsed twice with 500 μl of 70% ethanol (Carlo ERBA Reagents, Val-de-Reuil, France). DNA was re-suspended in 35 μl of TE buffer, and then quantified and checked for quality using a NanoDrop Spectrophotometer (Thermo Fisher Scientific, United States). DNA samples were then stored at −20°C until use.

#### Bacterial 16S rRNA Gene Sequencing

The hypervariable V4 region of the bacterial 16S rRNA gene was amplified by polymerase chain reaction (PCR) using the Earth Microbiome Project primer pair: 515F (Parada et al., 2016) and 806R (Apprill et al., 2015). PCR mixture was composed of 6.75 μl of nuclease-free water, 0.25 μl of Q5 High-Fidelity DNA Polymerase (2 U/μl) (New England Bioloabs, Ipswich, MA, United States), 5 μl of 5X Q5 High GC Enhancer, 5 μl of 5X Q5 Reaction Buffer, 1.25 μl of each primer at 10 μM, 0.5 μl of dNTP at 10 mM and 5 μl of sample DNA diluted at 10 ng/μl, for a final volume of 25 μl. The DNA template was amplified in the following thermal conditions: initial denaturation for 30 s at 98°C, followed by 30 cycles of 10 s at 98°C, 30 s at 50°C, 30 s at 72°C, and a final extension step at 72°C for 7 min. Each sample was amplified in triplicate and pooled into a single volume of 75 μl. PCR products were then cleaned-up, barcoded and normalized according to Illumina guidelines and the “16S Metagenomic Sequencing Library Preparation” protocol. Sequencing was performed with a MiSeq instrument (Illumina) with v3 chemistry and generated 300 bp paired-end reads, which were demultiplexed with Illumina run software.

#### Bioinformatic Processing of the Data

Demultiplexed reads (around 300 bp) were first quality-checked using FastQC (Andrews, 2010), and trimmed with FASTX-trimmer from the FASTX-Toolkit (Hannon, 2010) as follows: reads R1 were trimmed after 280 bp and reads R2 after 230 bp. Reads were then processed using the FROGS pipeline (Escudié et al., 2018). Reads were merged using Flash (Magoè and Salzberg, 2011) with 10% mismatches authorized in overlap regions, and primer sequences removed with Cutadapt (Martin, 2011). Merged reads were clustered using Swarm (Mahé et al., 2014) as recommended by Escudié et al. (2018), with a first execution with an aggregation parameter equal to 1, followed by a second execution on previous cluster seeds with an aggregation parameter equal to 3. Chimera detection and removal was performed using VSEARCH (Rognes et al., 2016). Clusters were then filtered on abundance and occurrence by representing a minimum 0.005% of all sequences and being present in at least three samples (referring to the number of biological replicates). Cluster affiliation was performed with blastn+ (Camacho et al., 2009) against the 16S Silva database version 123 (Quast et al., 2013), and OTUs (operational taxonomic units) were filtered depending on an identity and coverage value of 100%.

Downstream analyses were performed on rarified counts with R version 3.4.4 (R Core Team, 2018) under RStudio environment version 1.1.442 (RStudio Team, 2016). Alpha-diversity indexes (Observed and Shannon) and β-diversity were computed using the R packages phyloseq (McMurdie and Holmes, 2013). Beta-diversity analysis was done using the Weighted UniFrac distance, and samples structure was visualized with a MDS ordination plot. The R package DESeq2 (Love et al., 2014) was used to perform differential abundance analysis on samples raw counts normalized following a rlog transformation in base 2 with a pseudo-count of 1. In these samples, prior to the normalization, only OTUs presenting a sequence number greater than 0.05% of the sum of all sequences were kept. All graphical visualizations were performed with the R package ggplot2 (Wickham, 2009).

#### Deposit and Accessibility of Sequences

The raw fastq formatted data were deposited on Ifremer’s Sextant database and are accessible through DOI number: https://doi.org/10.12770/52b1f566-cafb-4d91-9acc-491386a58a46.

### Salmon Gravlax Sensory Evaluation

Sensory evaluation of salmon gravlax on Batch 1, free of *L. monocytogenes*, was performed in parallel to the microbiological analyses. For each condition (control and inoculated samples), and at each sampling interval (T0 to 25 days), a conventional sensory profiling test was conducted on gravlax slice odor, according to ISO 13299 (2003) Standard. The sensory evaluation was performed by an internal trained panel of 17 judges experienced in seafood, especially in salmon products (Joffraud et al., 2006; Macé et al., 2013). During the session, the panelists were asked first to assess global spoilage based on off-odor perception. They were then asked to characterize it using a list of relevant sensory descriptors specific to seafood spoilage (salmon products mainly) and PC sensory signature (Wiernasz et al., 2017). The sensory descriptors used were: butter, acid, sour, amine, feet/banana, sulfur, dill and fish odors. Both global spoilage and descriptors were scored for intensity on a continuous scale from 0 (low intensity) to 10 (high intensity). The product was considered strongly spoiled and unfit for consumption when the overall spoilage level exceeded a threshold value of 6.

Sessions were performed in individual partitioned booths, as described in procedure NF V-09-105 (ISO 8589, 2010) and equipped with a computerized system (Fizz, Biosystèmes, Couternon, France). For the practical part, each panelist received one slice of salmon gravlax presented in a covered plastic container. Samples were assigned three-digit numbers and randomized for order presentation within the panelists. A normalized principal component analysis (PCA) was performed on sensory descriptor mean scores using the R package ggfortify (Horikoshi and Tang, 2016).

### Biochemical Analysis

#### Physical and Chemical Parameters

Total volatile basic nitrogen (TVBN) and trimethylamine (TMA) were quantified, at T0 (control only) and after 14 days, from 80 g of minced product (Batch 1), according to Conway’s micro-diffusion method (Conway and Byrne, 1933). The pH value was measured directly after microbiological analyses in the stomached solution with a pH-meter (Mettler Toledo AG, Schwerzenbach, Switzerland).

#### Biogenic Amine Quantification

Biogenic amines concentrations were determined at T0 and after 14 and 21 days. 10 ml aliquots from stomached solution obtained from Batch 1 samples were taken and 5 mL of a 12% trichloroacetic acid solution (Panreac, Darmstadt, Germany) was added. Samples were kept frozen at −20°C until analysis. Three biogenic amines (putrescine, cadaverine, and tyramine) were quantified by high pressure liquid chromatography (HPLC) following the method of Wiernasz et al. (2017).

### Volatilome Investigation by Headspace SPME/GC-MS

For each sampling time, 20 g of gravlax (Batch 1) was withdrawn and stored in vacuum packaging at −40°C before analysis. Eight salmon flesh cylinders were sampled across the frozen product using a pre-cooled metal cork borer and immediately pooled to make up 1 g of analysis sample. Samples were kept frozen in 4 mL vials with screw caps and PTFE/silicone septa at −40°C prior to extraction and analysis. For each sample (time point and treatment), three independent analysis samples (triplicate) were prepared.

Prior to volatile extraction, a 30% w/v NaCl solution (H_2_O) was added to the sample, which was finally minced using a high-speed homogenizer. HS-SPME was applied for extraction of volatile organic compounds (VOCs) using a manual SPME holder with a PDMS/DVB-coated 65 μm fiber (Supelco Inc., Bellefonte, PA, United States). Prior to extraction, the SPME fiber was conditioned in the injection port of the GC according to the instructions provided by the supplier. The SPME fiber was exposed to the atmosphere in the closed sample vial for 30 min, while keeping the vial isothermally at 50°C in a water bath. Samples were agitated with a magnetic stirrer throughout the extraction.

An Agilent 6890/5975 GC-MS (Agilent Technologies Inc., Palo Alto, CA, United States) was used for all analyses. Analytes absorbed on the SPME fiber were desorbed in the injection port for 3 min under splitless conditions. GC separations were carried out using an apolar HP-5MS capillary column (30 m × 0.25 mm and film thickness 0.25 μm). Injection temperature was 220°C, and the interface was set to 220°C. The carrier gas was He at a constant flow rate of 1 ml/min. GC temperature was ramped from 40°C to 211°C at a rate of 4.5°C/min, then raised at a rate of 50°C/min and finally held at 220°C (total run time: 40 min). The MS source was adjusted to 230°C, and a mass range of *m/z* 35–350 was recorded. Mass spectra were acquired in electron impact ionization (EI) mode at 70 eV.

GC-MS chromatograms were visualized using the following GC-MS software packages: Agilent ChemStation software (Agilent Technologies, Waldbronn, Germany), AMDIS software (version 2.71; National Institute of Standards and Technology, Boulder, CO., United States), and the open source software OpenChrom Community Edition Alder (version 1.2.0) (Lablicate GmbH, Hamburg, Germany)^1^.

Tentative identification of compounds was carried out using (a) MS libraries such as NIST05 spectral library (National Institute of Standards and Technology, Gaithersburg, MD, United States), the NIST Chemistry WebBook^2^ and a customized in-house MS library of VOCs, in combination with (b) linear retention indices (LRI), based on an homologous series of even numbered n-alkanes (C8–C24), in combination with LRIs found in the literature and NIST Chemistry WebBook. GC-MS data integration, normalization (total signal) and alignment was carried out using Metalign software (PRI-Rikilt, Wageningen, Netherlands). Detected analyte concentrations were estimated quantitatively based on an internal standard (butylated hydroxytoluene, BHT) and expressed as μg/kg.

Multivariate analyses on VOC composition were performed by hierarchical clustering analysis (HCA) coupled with a heatmap on concentration means corrected by the median and transformed into log2(n). The HCA was carried out with the R package gplots (Warnes et al., 2016) using the Ward clustering method on Pearson correlation distance.

### Statistical Analyses

All statistical analyses were performed using the R package vegan version 2.4.6 (Oksanen et al., 2018). PCs TVBN and biogenic amine productions were compared to the controls at each sampling time using a one-way ANOVA followed by a *post hoc* HSD-Tukey multiple comparison test. Samples sensory global spoilage scores were compared at each sampling time by performing a one-way ANOVA followed by a *post hoc* HSD-Tukey multiple comparison test. Alpha-diversity difference between samples during time was also assessed using a one-way ANOVA and a *post hoc* HSD-Tukey multiple comparison test.

## Results

### PC Impact on Microbial Ecosystems

#### Cultural Microbial Analyses

**FIGURE 1:**
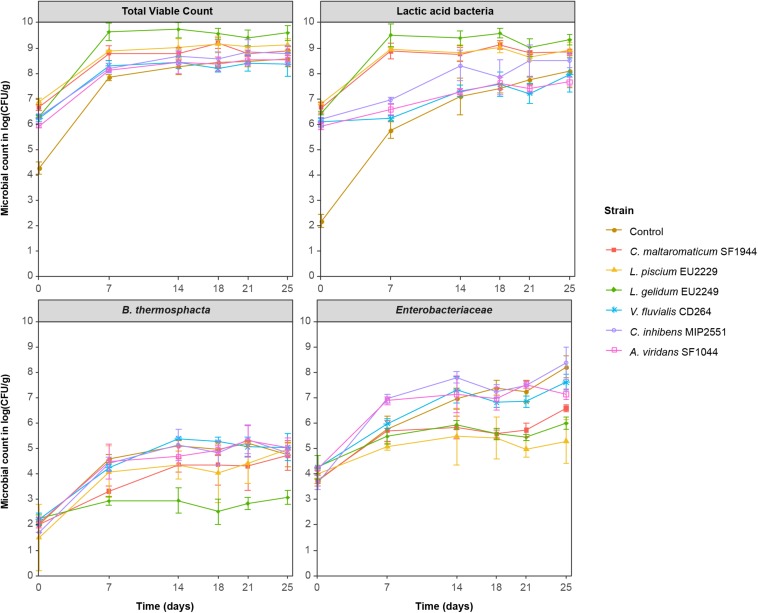
Salmon gravlax microbial groups evolution in the presence of the six protective cultures during storage at 8°C.

Growth kinetics of the four microbial groups during storage at 8°C in salmon gravlax Batch 1 are presented in Figure 1. For controls, at the beginning of the experiment, the TVC and LAB counts were 4.3 ± 0.2 and 2.2 ± 0.3 log CFU/g respectively. Subsequently, both microbial groups quickly grew and reached their maximum after 14 days of storage for TVC with 8.3 ± 0.3 log CFU/g, and after 25 days for LAB with 7.1 ± 0.7 log CFU/g. In the inoculated samples, the initial PC count (NAP and LH media) was close to the expected concentration, with values between 6.1 and 6.8 log CFU/g. By comparing TVC and LAB count, *C. maltaromaticum* SF1944, *L. piscium* EU2229 and *L. gelidum* EU2249 strains seemed to be more competitive than the 3 other strains in the product. Their maximum concentration, reached after 7 days, was higher with values between 8.9 and 9.5 log CFU/g. TVC and LAB counts were identical over time and always 1–2 log CFU/g higher than in controls, suggesting that these PCs remained dominant during storage. Conversely, in the presence of *V. fluvialis* CD264, *A. viridans* SF1044, and *C. inhibens* MIP2551, LAB counts reached their maximum concentration after 14 days and remained in a range of concentrations close to the control, never exceeding 7.9 log CFU/g. On LH medium, in their presence, the TVC reached its maximum after 7 days with concentrations 1.0 log CFU/g higher than the LAB counts, suggesting that these three PCs were less competitive.

In control, *Enterobacteriaceae* initial population was around 4.0 log CFU/g and reached its maximum concentration after 25 days of storage with a value of 8.2 ± 0.4 log CFU/g. *C. maltaromaticum* SF1944, *L. piscium* EU2229 and *L. gelidum* EU2249 inhibited their growth after 14 days and until the end of storage with a log(CFU/g) reduction of 1.6, 2.9, and 2.2 after 25 days respectively. In the presence of *V. fluvialis* CD264, *C. inhibens* MIP2551 and *A. viridans* SF1044, *Enterobacteriaceae* counts was not different from controls. An exception for *A. viridans* SF1044 was observed, which displayed a slight inhibition (1.1 log CFU/g) after 25 days.

*Brochothrix thermosphacta* initial concentration in gravlax was around 2.0 log CFU/g and never exceeded 5.1 ± 0.2 log CFU/g. A significant inhibitory effect was observed in the presence of *L. gelidum* EU2249, as the *B. thermosphacta* count was maintained below 3.0 log CFU/g throughout the experiment.

#### Microbial Composition Through Metabarcoding

A total of 8,430,429 raw reads were obtained after sequencing. An average of 61,087 reads per sample passed through the FROGS pipeline with a range from 6,895 up to 176,787 reads. All samples, except two replicates from the control condition at T0 (samples T2D0 and T3D0) with 6,895 and 9,674 reads respectively, were normalized by rarefaction on a read number of 14,375. These two replicates were kept unormalized only for ecosystem composition exploration and visualization and DESeq2 differential abundance analysis, and were systematically removed for α- and β-diversity calculation and statistical analyses.

**FIGURE 2:**
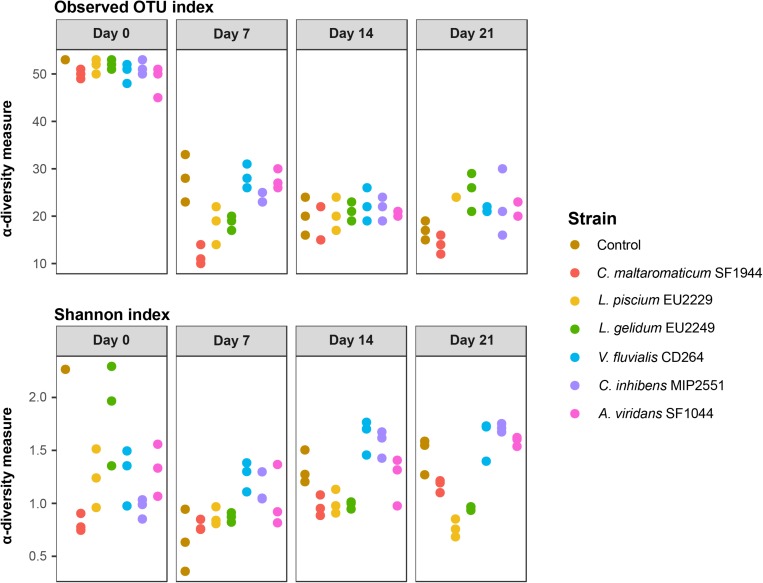
Microbial ecosystem α-diversity measures at each sampling time in the presence of the six protective cultures.

**FIGURE 3:**
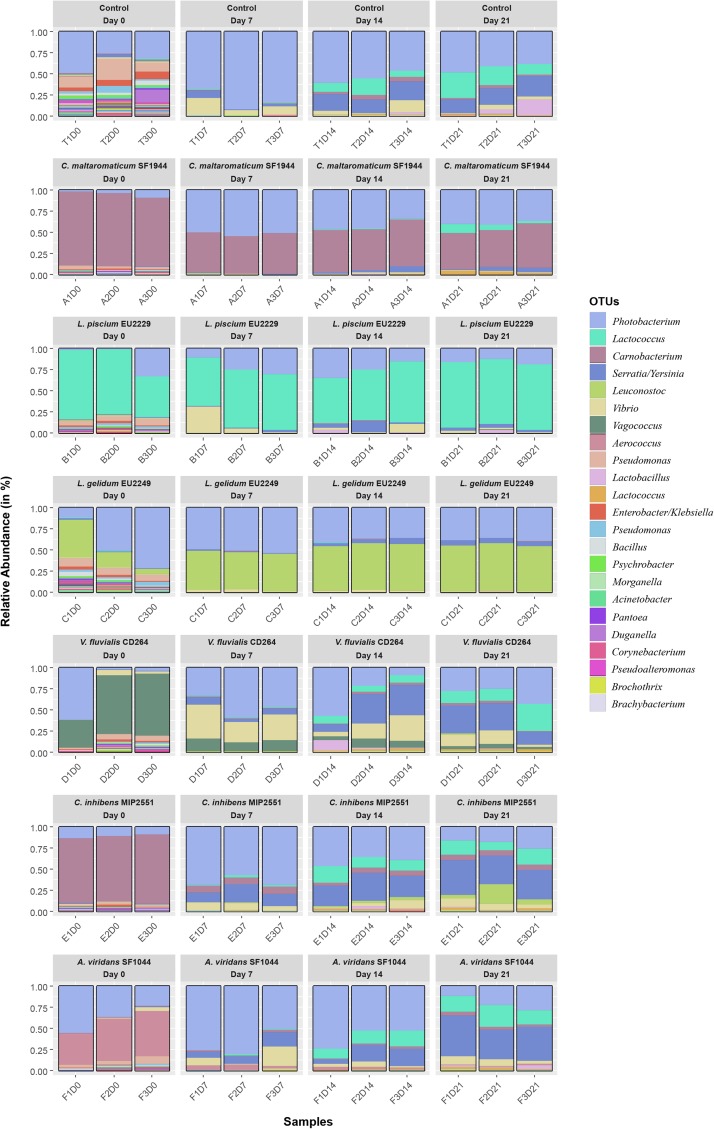
Salmon dill gravlax ecosystem relative composition in the presence of the six protective cultures. Only OTUs with a sequences number representing more than 0.1% of total reads number are presented in the legend. Also see Supplementary Data 1.

In controls at T0, diversity was high with 53 OTUs observed (Figure 2). The ecosystem was dominated mainly by *Photobacterium* (49%) and *Pseudomonas* (13%), but also by many subdominant OTUs representing each 0.5–2.5% of the total composition (Figure 3). After 1 week of storage, this number decreased drastically and significantly (*p*-value < 0.05) to 28 OTUs on average (Figure 2). The ecosystem was then largely dominated by *Photobacterium* (68–92%), *Vibrio* (21–56%) and *Serratia/Yersinia* (1–8%) to a lesser extent, while *Pseudomonas* abundance decreased to less than 0.5% of the total composition.

After 14 days of storage, the microbial diversity in the control decreased significantly to finally reach a minimum of 17 OTUs observed after 21 days. At day 14 the ecosystem composition was still dominated by *Photobacterium* (38–60%), *Serratia/Yersinia* (16–23.5%), *Vibrio* (3–14%). In addition, *Lactococcus* and *Carnobacterium* increased significantly (DESeq2 pairwise differential abundance analysis, *p*-value < 0.05) as well (Figure 3), representing respectively 7.5 to 29.5% and 2 to 5% depending on the replicate. Between 14 and 21 days, ecosystems composition was almost identical. Only *Lactobacillus* and *Aerococcus* OTUs, appeared to be more abundant at Day 21 (*p*-value < 0.05), representing respectively 0.5–18.5% and 0.5% of the total composition depending on the replicate.

For the inoculated samples, the OTUs corresponding to the PC genera were largely dominant within the microbiota and significantly more abundant than in controls at T0 (Figure 3). These OTUs were therefore used to follow the PC implantation during storage time in the product. Considering that the hyperviable region V4 of the 16S rRNA gene was not discriminant enough to distinguish between the two *Carnobacterium* species, the OTU corresponding to *Carnobacterium* genus was used to follow the PCs in the samples inoculated with *C. maltaromaticum* SF1944 and *C. inhibens* MIP2551.

At T0, despite a 2.0 log CFU/g difference in TVC between inoculated samples and control (Figure 1), no statistical differences were observed for α-diversity (*p*-value ≥ 0.05). After 7 days, like in the controls, diversity drastically fell, with 11–28 OTUs observed (Figure 2). Up to 21 days of storage no significant evolution in diversity was observed within samples (*p*-value ≥ 0.05).

From Day 7, two scenarios were observed (Figure 3). The first one corresponded to the samples inoculated with *V. fluvialis* CD264, *C. inhibens* MIP2551 and *A. viridans* SF1044. The abundance of their corresponding OTUs decreased drastically after 7 days. However, they remained present over time among the minority OTUs, with constant proportions varying between 0.7 and 13% (Figure 3). Except for *C. inhibens* MIP2551, the only statistical difference in composition overtime from the controls was the presence of the bioprotective strain itself and a higher proportion of *Leuconostoc* in all samples (Supplementary Data 2).

In the second scenario, *C. maltaromaticum* SF1944, *L. piscium* EU2229 and *L. gelidum* EU2249 grew well in the product and remained largely dominant until the end of the experiment, representing more than 50% of the total ecosystem. In addition, *Photobacterium* and *Serratia/Yersinia* were also major genera throughout the storage representing respectively 15–50% and 1.7–14% of the ecosystem. By comparing the microbial ecosystem in the presence of these three PC strains with controls by a pairwise differential abundance analysis at each sampling time, only few differences in composition were recorded (including the strain itself) (Supplementary Data 2). These results suggest that, though largely dominant, *C. maltaromaticum* SF1944, *L. piscium* EU2229 and *L. gelidum* EU2249 had only a slight effect on the gravlax natural microbial ecosystem, at least on the most abundant OTUs.

**FIGURE 4:**
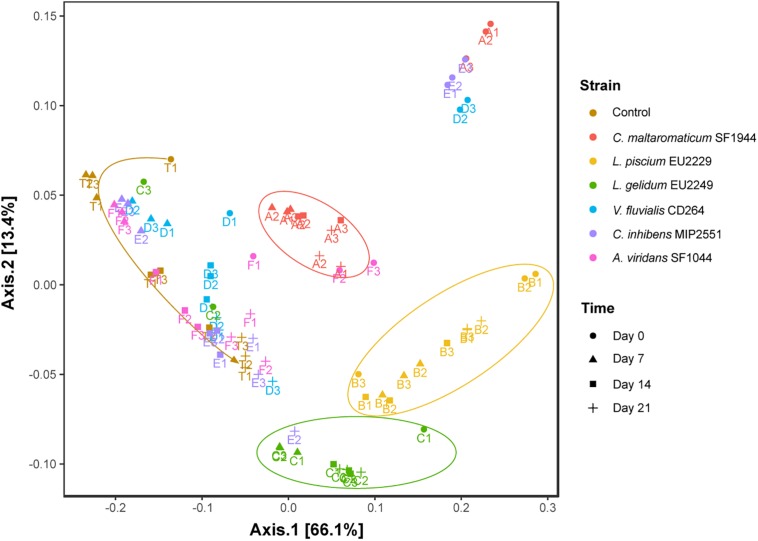
Two-dimensional scaling representation of sample microbial composition on dimension 1–2, based on weighted UniFrac distance. A: *C. maltaromaticum* SF1944, B: *L. piscium* EU2229, C: *L. gelidum* EU2249, D: *V. fluvialis* CD264, E: *C. inhibens* MIP2551, F: *A. viridans* SF1044 and T: Control. Numbers following letters indicate the replicate number.

These two scenarios in the presence of the PC strains are summarized in Figure 4 showing the distance between samples on an MDS ordination plot using the weighted UniFrac distance. Samples inoculated with *C. maltaromaticum* SF1944, *L. piscium* EU2229 and *L. gelidum* EU2249 were respectively tightly clustered together, while samples inoculated with *V. fluvialis* CD264, *C. inhibens* MIP2551 and *A. viridans* SF1044 followed the exact same evolution as the control.

### PC Antilisterial Activity

**FIGURE 5:**
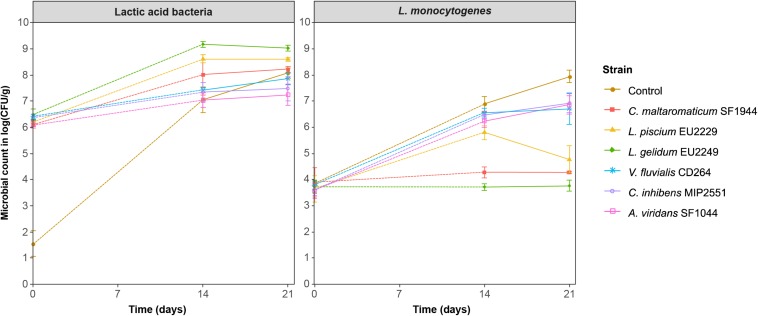
*Listeria monocytogenes* (cocktail of strains RF191, RF107, RF114, RF131, and RF151) growth in salmon gravlax in the presence of the six protective cultures during 3 weeks of storage at 8°C.

Figure 5 summarized the antilisterial activity of the six PC on salmon gravlax against a cocktail of five *L. monocytogenes* strains.

Like for Batch 1, the initial PC concentrations were close to the expected counts with values ranging from 6.1 to 6.5 log CFU/g, and *C. maltaromaticum* SF1944, *L. piscium* EU2229 and *L. gelidum* EU2249 were more competitive in the product than the three other PCs. The initial level of *L. monocytogenes* inoculated in samples ranged from 3.6 to 3.9 log CFU/g and reached 7.9 ± 0.2 log CFU/g in the control after 21 days of storage.

During this experiment, three inhibition patterns against *L. monocytogenes* were observed: (i) *C. maltaromaticum* SF1944 and *L. gelidum* EU2249 totally prevented the growth of *L. monocytogenes* during storage, maintaining it at its initial concentration (bacteriostatic effect); the inhibitions were 4.1 and 3.6 log CFU/g after 21 days for *C. maltaromaticum* SF1944 and *L. gelidum* EU2249 respectively, (ii) *L. piscium* EU2229 strain also displayed a strong inhibition, with 3.1 log CFU/g reduction at Day 21, but with different behavior; a bactericidal effect appeared after 14 days when the PC reached its maximum population; *L. monocytogenes* count decreased from 5.8 ± 0.3 at Day 14, to 4.8 ± 0.5 log CFU/g at Day 21, and (iii) in the presence of *V. fluvialis* CD264, *C. inhibens* MIP2551 and *A. viridans* SF1044 a slight inhibition (1.0 log CFU/g) was visible only after 21 days of storage.

### Impact on Salmon Dill Gravlax Organoleptic Properties

**FIGURE 6:**
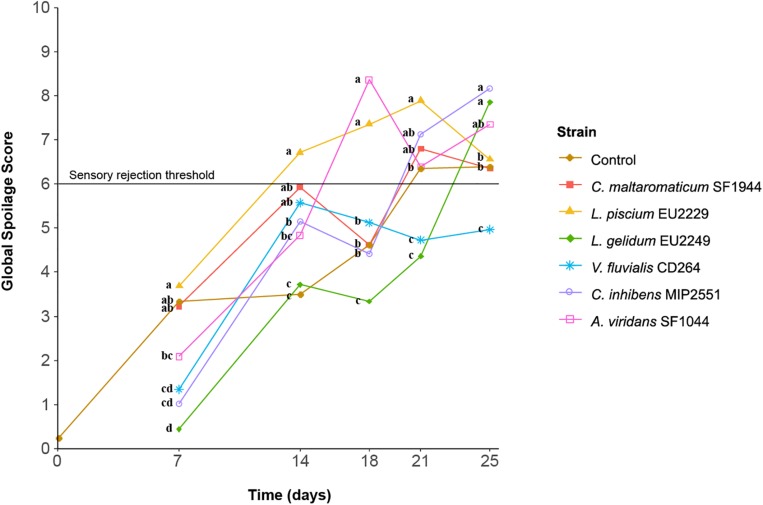
Sensory global spoilage score of salmon gravlax slices inoculated with the six protective cultures. Samples associated with identical letters are not significantly different, according to a HSD-Tukey test with a *p*-value of 0.05 performed after an ANOVA.

**FIGURE 7:**
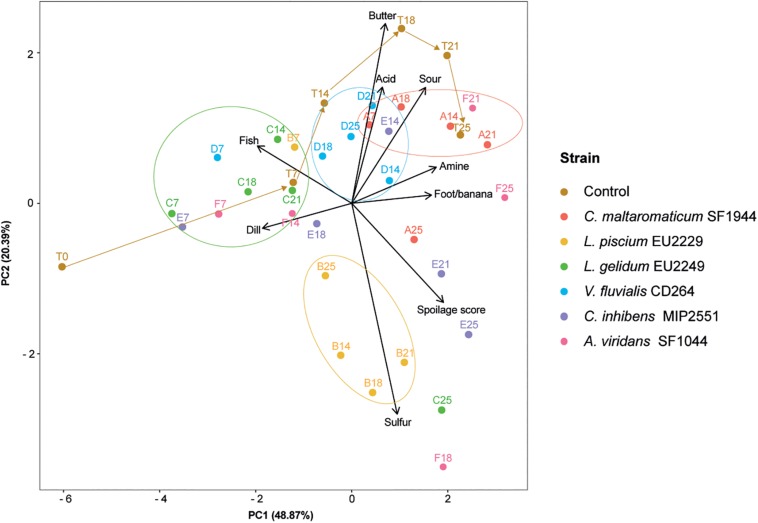
Normalized principal component analysis (PCA) representation in dimension 1–2 of the salmon dill gravlax sensory descriptors in the presence of the six protective cultures. The PCA is based on mean scores of each descriptor. A: *C. maltaromaticum* SF1944, B: *L. piscium* EU2229, C: *L. gelidum* EU2249, D: *V. fluvialis* CD264, E: *C. inhibens* MIP2551, F: *A. viridans* SF1044 and T: control, numbers following letters refer to the sampling time.

Control gravlax quickly showed signs of spoilage with an overall spoilage score of 3.3 after 1 week of storage and was rejected by panelists after 21 days with a score of 6.3 (Figure 6). The sensory profiles illustrated on the PCA in Figure 7 reveal a progression from strong fish and dill odor at the beginning of the storage to amine, acid, and sour odors at Day 21. A strong butter-like odor was also recorded at 18 days but not later.

Gravlax slices inoculated with *L. piscium* EU2229 were rapidly spoiled and rejected by panelists after only 14 days (score of 6.7) due to marked sulfur off-odors, then acid and amine smells after leaving the plastic containers opened for few seconds. In addition, from Day 14, salmon gravlax slice color turned from orange to pink, with a cooked-like appearance.

Samples inoculated with *A. viridans* SF1044 also exceeded the sensory threshold between 14 and 18 days with strong amine odor combined with sulfur and sour smells.

In the presence of *C. maltaromaticum* SF1944, the spoilage score was not different from controls except at 14 days when it reached a higher score of 5.9. On the PCA, samples inoculated with this strain were clustered together and characterized by odors such as sour, amine and feet/banana or malty/rhubarb depending on the panelists’ perception.

Samples inoculated with *C. inhibens* followed a similar pattern, but with a higher spoilage score at Days 21 and 25, and a sulfur smell production.

The inoculation with *L. gelidum* EU2249 and *V. fluvialis* CD264 seemed to limit sensorial spoilage. For the *Leuconostoc* strain, the global spoilage remained low from 7 days (0.4) to 18 (3.3) and was still acceptable after 21 days (4.4), with fatty fish and dill odors still strongly perceived and a slight acid smell. However, from Day 14, the product had a pink cooked-like appearance and a slight formation of gas and yellow slime were visible in the package. In the case of *V. fluvialis* CD264 the spoilage score remained under the threshold during the whole storage period of 25 days. Similar to the controls, gravlax slices inoculated with this strain were characterized by amine, sour and acid off-odors, but at lower intensity.

### Biochemical Changes

#### Physical and Chemical Parameters

**FIGURE 8:**
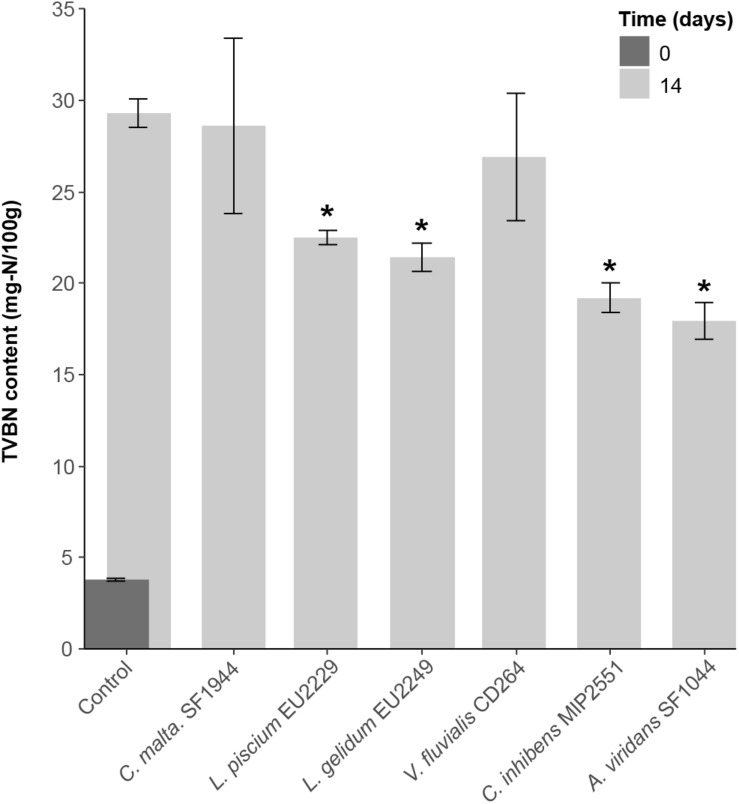
Total volatile basic nitrogen (TVBN) production after 14 days of storage in the presence of the six protective cultures. Values followed by ^∗^ are significantly different from the control value with a *p*-value of 0.05.

In controls, at the beginning of the experiment, the TVBN content was very low (3.8 ± 0.1 mg-N/100 g) (Figure 8). After 14 days, it increased and reached 29.3 ± 0.8 mg-N/100 g. In comparison, in samples inoculated with *L. piscium* EU2229, *L. gelidum* EU2249, *C. inhibens* MIP2551, and *A. viridans* SF1044 a significant reduction in TVBN content was observed (*p*-value < 0.05). No TMA was detected at Day 0 and less than 5 mg-N/100 g was recorded after 14 days in controls or in samples inoculated with the six PCs.

**FIGURE 9:**
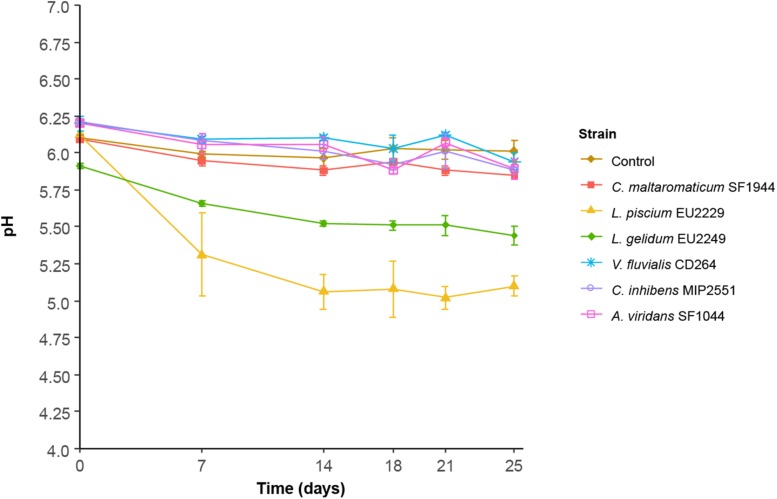
Salmon dill gravlax pH evolution during storage in the presence of the six protective cultures.

The pH was stable throughout the storage period, with values of around 6.0 in controls and in samples inoculated with *C. maltaromaticum* SF1944, *C. inhibens* MIP2551, *V. fluvialis* CD264 and *A. viridans* SF1044 (Figure 9). *L. piscium* EU2229 and *L. gelidum* EU2249 induced a rapid acidification of the product and the pH reached minimum values of 5.0 ± 0.1 and 5.5 ± 0.02 respectively, after 14 days of storage.

#### Biogenic Amine Content

**FIGURE 10:**
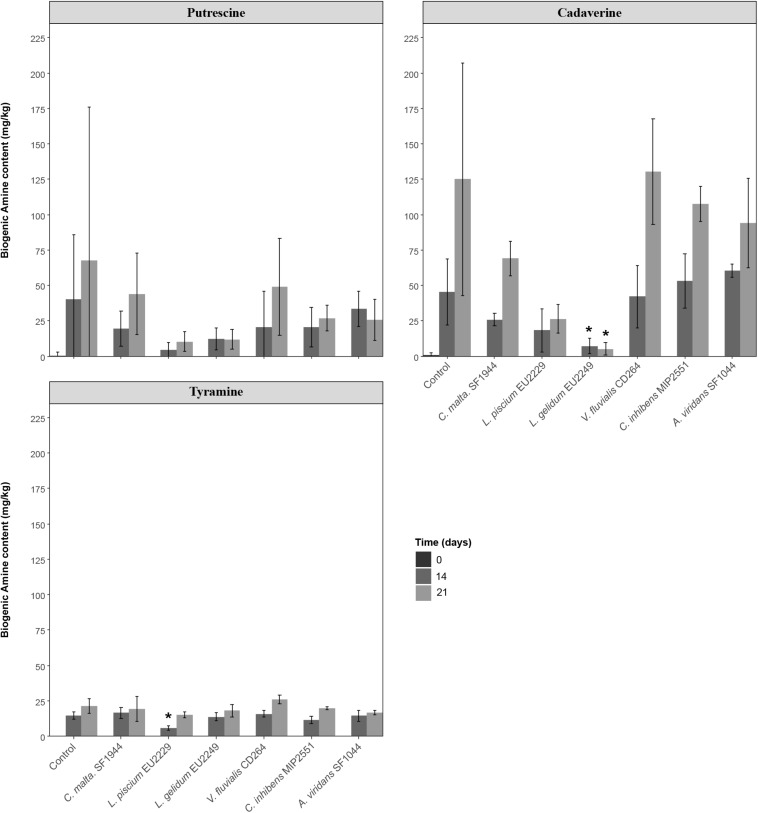
Putrescine, cadaverine and tyramine content in salmon gravlax in the presence of the six protective cultures. Values followed by ^∗^ are significantly different from the control value with a *p*-value of 0.05.

Variability among samples was marked, especially for putrescine and cadaverine (Figure 10). In controls, biogenic amine production increased during storage and reached concentrations of 67 ± 108, 125 ± 82, 21 ± 5 mg/kg after 21 days, respectively, for putrescine, cadaverine and tyramine. A significant reduction of the cadaverine content was noted at Days 14 and 21 only in the presence of *L. gelidum* EU2249. A significantly lower concentration of tyramine was also observed after 14 days only in the presence of *L. piscium* EU2229.

### Volatilome Composition

VOCs were measured by HS-SPME/GC-MS analysis on the first day of the experiment for the controls and for all samples after 14 and 21 days. In all, 100 VOCs were detected among which 50 were identified, 46 were identified structurally and by chemical class and four remained unknown (Supplementary Data 3).

**FIGURE 11:**
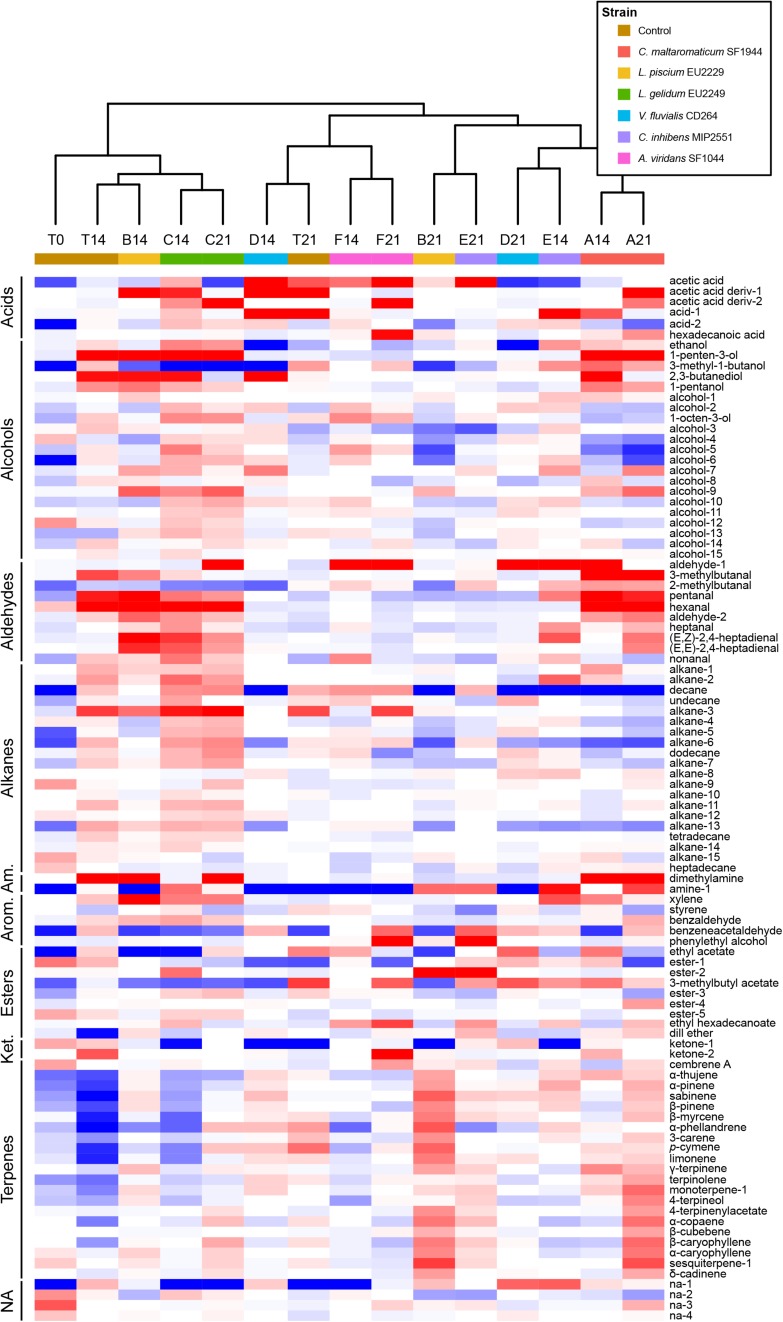
Hierarchical cluster analysis (HCA) heatmap of control and protective culture volatilome composition, based on Pearson correlation calculated from log2(n) transformation of the median based ratio on VOCs mean concentrations (*n* = 3). Bluish colors indicate lower metabolite concentrations, reddish colors show higher metabolite level. Am, Amines; Arom, Aromatics; Ket, Ketones.; NA, Not identified. A: *C. maltaromaticum* SF1944, B: *L. piscium* EU2229, C: *L. gelidum* EU2249, D: *V. fluvialis* CD264, E: *C. inhibens* MIP2551, F: *A. viridans* SF1044 and T: Control. Numbers following letters refer to the sampling time. See Supplementary Data 3.

Sample volatilome composition and evolution were analyzed further by HCA analysis coupled with a heatmap visualization (Figure 11). The volatilome profile appeared specific for the controls and each inoculated strain. The higher diversity of the volatilome profile was observed in presence of *L. gelidum* EU2249 whereas it was quite low for samples inoculated with *V. fluvialis* CD264, *A. viridans* SF1044 and *C. inhibens* MIP2551. No major modifications of volatilome composition were observed between 14 and 21 days when gravlax were inoculated with *C. maltaromaticum* SF1944, *A. viridans* SF1044 and *L. gelidum* EU2249. On the opposite, strong differences were observed between sampling times for the controls and the three other strains. As example, samples containing *V. fluvialis* CD264 were characterized by a high 2,3-butanediol production with a concentration of 70855 ± 6671 μg/kg (versus 1475 ± 45 μg/kg in control) at T14 whereas the concentration of this compound decreased drastically and was similar to the control at T21 (Supplementary Data 3).

Some specific compounds seemed to be associated with the presence of some inoculated bacteria at the end of the storage (21 days) by comparison to the non-inoculated control. In presence of *C. maltaromaticum* SF1944 at T21, samples were mainly characterized by an increase of dimethylamine, 3-methylbutanal, pentanal, hexanal and 1-penten-3-ol. When compared to all the strains and to the controls, this strain was the strongest 3-methybutanal producer during storage with a concentration of 8,262 ± 1,499 μg/kg at 14 days and 4,695 ± 581 μg/kg at 21 days (respectively 9 and 27 times higher than in controls) (Supplementary Data 3). The presence of *L. piscium* EU2229 induced higher concentrations of many terpenes (sabinene, sesquiterpene-1, 4-terpinenylacetate, β-pinene, limonene, α-caryophyllene). In samples inoculated with *L. gelidum* EU2249 and *A. viridans* SF1044, a marked increase in acetic acid (derivative-2) was observed after 21 days. Dimethylamine, hexanal, 1-pentene-3-ol, and ethanol were also produced in higher quantities in the presence of *L. gelidum* EU2249 than in the controls. The samples inoculated by *V. fluvialis* CD264, *C. inhibens* MIP2551 and *A. viridans* SF1044 were characterized by a higher content of benzeneacetaldehyde at T21 compared to the controls.

## Discussion

In this study, the impact of six bioprotective LABs on the salmon gravlax quality and safety during cold storage, was evaluated in a polyphasic approach: microbial ecosystem (cultural method and metabarcoding approach), physical and chemical parameters, volatilome composition and sensory properties. Based on the PC strains considered, two scenarios were highlighted.

The first scenario concerns *C. maltaromaticum* SF1944, *L. piscium* EU2229 and *L. gelidum* EU2249 which grew quickly in the product and remained largely dominant in the microbial ecosystem throughout storage. These three species are known to be well-adapted to seafood and meat products, as they are commonly isolated and often found as dominant members of the microbiota in many matrices at different storage times (Afzal et al., 2010; Leroi, 2010; Ghanbari et al., 2013; Pothakos et al., 2014b; Chaillou et al., 2015; Saraoui et al., 2016b; Zagorec and Champomier-Vergès, 2017).

*Carnobacterium maltaromaticum* SF1944 and *L. gelidum* EU2249 totally prevented the growth of *L. monocytogenes* during the 21-day trial period. The antimicrobial activity of *Carnobacterium* species and *L. gelidum* against *L. monocytogenes* in seafood is largely described (Duffes et al., 1999a, b; Brillet et al., 2004, 2005; Nilsson et al., 2004; Tahiri et al., 2004, 2009; Vescovo et al., 2006; Matamoros et al., 2009; Saraoui et al., 2017). *Carnobacterium* and *Leuconostoc* species are known to be able to produce a wide range of bacteriocins active against many close related Gram-positive bacteria including many species of LAB and *L. monocytogenes* (Leisner et al., 2007; Afzal et al., 2010; Pilet and Leroi, 2011; Woraprayote et al., 2016).

*Lactococcus piscium* EU2229 also displayed antimicrobial activity against *L. monocytogenes* (3.1 log CFU/g reduction), occurring only after 14 days of storage, when the PC strain reached its maximum concentration. Such behavior was already described for *L. piscium* CNCM I-4031 on shrimp; its mechanism of action seems to be cell-contact-dependent and induced by quorum sensing (Fall et al., 2010b; Saraoui et al., 2016a, 2018).

These three PC strains displayed an antimicrobial activity against *Enterobacteriaceae* during storage. In the case of *L. gelidum* EU2249 and *L. piscium* EU2229, this activity could be linked to the product acidification that was recorded from Day 7. Although *Carnobacterium* species are not particularly known to be active against Gram-negative bacteria, Brillet et al. (2005); Matamoros et al. (2009), and Saraoui et al. (2017) have shown that *C. alterfunditum* EU2257 and *C. divergens* V41 were able to inhibit *Enterobacteriaceae* by 2.0 log CFU/g on cooked and peeled shrimp and cold-smoked salmon stored under MAP and vacuum, respectively.

Despite their potentially useful antimicrobial activity against *L. monocytogenes* or other bacterial groups, the study of PC effect on sensory properties is crucial for their potential application.

Like for many LABs, the spoilage activity of *C. maltaromaticum* seems to be strain- and matrix-dependent (Leisner et al., 2007; Afzal et al., 2010). *C. maltaromaticum* was found to be responsible for the spoilage of cooked and peeled shrimp and sterile raw salmon (Mejlholm et al., 2005; Jaffrès et al., 2011; Macé et al., 2013, 2014), while no spoilage effect were found in cold-smoked salmon (Leroi et al., 1996; Duffes et al., 1999a; Brillet et al., 2005; Joffraud et al., 2006; Vescovo et al., 2006; Matamoros et al., 2009). In our study, the presence of *C. maltaromaticum* SF1944 did not induce any strong spoiling activity compared to the controls. The samples at the end of storage were characterized by malty sensory notes, typical of *C. maltaromaticum*, due to the production of 2-methylbutanal and 3-methylbutanal from leucine and isoleucine catabolism (Leisner et al., 2007; Afzal et al., 2010), found in high concentration in our inoculated samples.

When inoculated on naturally contaminated or sterile matrices, such as cooked and peeled shrimp, raw and cold smoked salmon, *L. piscium* did not induce off-odors (Matamoros et al., 2009; Fall et al., 2010a; Macé et al., 2013; Leroi et al., 2015; Saraoui et al., 2017). However, in gravlax, *L. piscium* EU2229 induced a strong production of sulfur, amine and acid smell. Although some isolates of *L. piscium* have been associated with a very weak sulfur smell production (Wiernasz et al., 2017), sulfur related off-odors in seafood are more likely linked to the metabolism of Gram-negative bacteria such as *H. alvei*, *S. liquefaciens*, *S. putrefaciens*, or some LABs such as *L. sakei* and *L. farciminis* (Leroi et al., 1998; Paludan-Müller et al., 1998; Stohr et al., 2001; Joffraud et al., 2006; Jaffrès et al., 2011; Macé et al., 2013, 2014). In our case, sulfur odor production induced by the presence of *L. piscium* EU2229 may have involved metabolism interactions with the gravlax natural microbiota.

Similarly, *L. gelidum* is not usually associated with seafood spoilage (Matamoros et al., 2009; Leroi et al., 2015). In fish juice, among 35 LABs from different species, *L. gelidum* strains were the most neutral compared to the controls regarding odor (Wiernasz et al., 2017). In vacuum-packed cooked and peeled shrimp, *L. gelidum* EU2247 improved sensory shelf-life after 28 days of storage (Matamoros et al., 2009). However, in gravlax, *L. gelidum* EU2249 induced from Day 14 a gas and slime formation in the package, and, after 25 days, vinegar and sulfur odors. As *Leuconostoc* are heterofermentative LABs and can be strong producers of acetic acid, CO_2_ and dextrans from sucrose (Cogan and Jordan, 1994; Björkroth and Holzapfel, 2006), the presence of residual sugar in gravlax may explain this spoilage effect. A similar case of exopolysaccharide production by *L. gelidum* and *L. gasicomitatium*, but in much higher quantity, has already been described in acetic-acid marinated herring (Lyhs et al., 2004).

The volatilome composition and evolution during salmon gravlax storage in controls was characterized by an increase in the concentrations of many alcohols, aldehydes, alkanes, acids and dimethylamine. Such types of volatile compounds, resulting mostly from microbial metabolic activity, increase simultaneously with deterioration of seafood organoleptic properties (Jørgensen et al., 2001; Olafsdóttir et al., 2005; Varlet et al., 2006; Jónsdóttir et al., 2008; Parlapani et al., 2014, 2015). Aldehydes, deriving mainly from microbial lipid oxidation, are especially good indicators of food degradation, and actively participate in the rancid, cooked-potatoes, fatty, floral, fruity and grassy odors of spoiled fish (Jørgensen et al., 2001; Varlet et al., 2007; Jónsdóttir et al., 2008).

In the presence of *L. piscium* EU2229, *C. maltaromaticum* SF1044 and *L. gelidum* EU2249 the volatilome composition during storage was different from controls and appeared to be species-specific. Some compounds produced by these bacteria on gravlax have already been reported in other food matrices inoculated with the same LAB species, such as 2- and 3-methylbutanal, 3-methylbutanol for *C. maltaromaticum* in shrimp (Jaffrès et al., 2011), 3-methyl-1-butanol produced by *L. piscium* in shrimp Fall et al. (2010a), and ethanol and acetic acid produced by *L. gelidum* in blood sausages, beef, sweet bell peppers and boiled eggs (Pothakos et al., 2014a). Other components such as terpenes, observed in high concentrations in samples inoculated with *L. piscium* EU2229 and *C. maltaromaticum* SF1944, are not currently described on the volatilome of these bacteria. Their presence may be linked to spice content of gravlax (dill and black pepper) but compared to the controls, it is likely that the PC metabolism plays a role in their production. Terpene synthesis and degradation by bacteria have been little studied. However, a recent genome-based analysis has evidenced the presence of predicted terpenes synthases in many bacterial orders (Yamada et al., 2015). Belviso et al. (2011) were the first authors to describe the ability of five LAB strains, isolated from cheese, to degrade α-campholenal and to produce a monoterpenoid isomer of borneol.

The second scenario concerns *V. fluvialis* CD264, *C. inhibens* MIP2551 and *A. viridans* SF1044, which were not competitive despite an initial inoculum level of 10^6^ CFU/g. Very soon after inoculation and until the end of storage, these strains collapsed in favor of the natural microbiota of the product and remained as a minority part of the ecosystem. In addition, they did not display antimicrobial activity, except a slight inhibition against *L. monocytogenes* after 21 days of storage. However, during their selection, these three strains totally inhibited all targets tested (*L. monocytogenes*, *H. alvei*, *P. phosphoreum*, *S. baltica*, *S. proteamaculans* and *L. sakei*) except for *B. thermosphacta* (Wiernasz et al., 2017). Afterward, it was shown that their broad-spectrum antimicrobial activity mostly relied on a large production of H_2_O_2_ (data not shown), which may not have been produced in our case in the absence of oxygen due to vacuum storage.

The sensory profile of the gravlax, in the presence of *C. inhibens* MIP2551 and *A. viridans* SF1044, was not very different from the controls. The spoilage was mainly characterized by amine and slight acid and sour odors, which are typically associated with raw salmon and cold-smoked salmon spoilage (Cardinal et al., 2004; Macé et al., 2012). This sensory profile may be correlated with the presence of *Photobacterium* which was largely dominant within the microbial ecosystem of these samples and is known to produce such spoilage odor in salmon products (Stohr et al., 2001; Macé et al., 2013). After 21 days of storage *C. inhibens* MIP2551 was also associated with a strong sulfur odor, also found for *A. viridans* SF1044, in addition to a light cheese/feet smell. Interestingly, *V. fluvialis* CD264 significantly reduced the global spoilage score and was the only PC strain able to extend salmon gravlax sensory shelf-life. The volatilome composition of the samples inoculated with these three PCs differs markedly from the controls, as relatively few metabolites were produced in high concentration for both sampling times.

Other quality parameters usually used as spoilage indicators such as biogenic amines (especially cadaverine and putrescine) (Jørgensen et al., 2000; Biji et al., 2016; de la Torre and Conte-Junior, 2018) and TVBN were not correlated to sensorial spoilage in the control gravlax samples. Putrescine and cadaverine levels remained at around 125 mg/kg, and the presence of PC strains did not impact their concentration. If ingested in high concentrations (100–800 mg/kg), tyramine may cause headaches and hypertensive effects in individuals deficient in monoamine oxidase (ten Brink et al., 1990; Halász et al., 1994). As *C. maltaromaticum* SF1944 and *V. fluvialis* CD264 were known to be large tyramine producers (Wiernasz et al., 2017) their content in salmon gravlax was also measured, but they never exceeded 25 mg/kg after 21 days.

## Conclusion

The effect of the six PCs on salmon dill gravlax quality could be classified in two main scenarios. The first scenario includes *C. maltaromaticum* SF1944, *L. piscium* EU2229 and *L. gelidum* EU2249, which were competitive in the product and dominated the ecosystem until the end of the experiment. They displayed antimicrobial activity against common spoilage bacteria and against *L. monocytogenes*. They also expressed their own sensory signatures and produced many volatile compounds. *L. piscium* EU2229 and *L. gelidum* EU2249 do not seem suitable for gravlax biopreservation as they both strongly acidified the product and were responsible for off-odors or slime and gas production. By contrast, *C. maltaromaticum* SF1944, induced no additional spoilage compared to controls, and so may be suitable for preserving salmon gravlax microbial safety by its antilisterial activity.

The second scenario includes *V. fluvialis* CD264, *C. inhibens* MIP2551, and *A. viridans* SF1044, which were not competitive and quickly collapsed in favor of the gravlax natural microbiota. They did not demonstrate strong antimicrobial activity and did not produced many volatile compounds. However, among these three strains, *V. fluvialis* CD264 maintained the salmon gravlax sensory quality below the rejection threshold even after 25 days and so may be a promising strain to improve gravlax quality during storage.

This work suggests that biopreservation of naturally contaminated products remains a complex matter and may rely more on metabolic interactions between microorganisms in an ecosystem than on antimicrobial activity alone.

## Data Availability Statement

The datasets generated for this study can be found in the 10.12770/52b1f566-cafb-4d91-9acc-491386a58a46.

## Author Contributions

NW, FL, and M-FP wrote the manuscript. NW, M-FP, DP, and FL designed the experiments. NW and FC were in charge of the experiments. NW worked on data processing and output visualizations. NW and MC carried out the statistical analyses. JC and MC worked on the sensory analyses. JR was responsible for the volatilome analyses. NW and SS worked on the extraction and amplicon sequencing.

## Conflict of Interest

SS was employed by the company Matıs, Research and Innovation, Exploitation and Utilization of Genetic Resources, Reykjavik, Iceland. The remaining authors declare that the research was conducted in the absence of any commercial or financial relationships that could be construed as a potential conflict of interest.

## Footnotes

1 https://www.lablicate.com

2 https://webbook.nist.gov/chemistry
